# Retrospective Analysis of INRG Clinical and Genomic Factors for 605 Neuroblastomas in Japan: A Report from the Japan Children’s Cancer Group Neuroblastoma Committee (JCCG-JNBSG) †

**DOI:** 10.3390/biom12010018

**Published:** 2021-12-23

**Authors:** Miki Ohira, Yohko Nakamura, Tetsuya Takimoto, Atsuko Nakazawa, Tomoro Hishiki, Kimikazu Matsumoto, Hiroyuki Shichino, Tomoko Iehara, Hiroki Nagase, Takashi Fukushima, Akihiro Yoneda, Tatsuro Tajiri, Akira Nakagawara, Takehiko Kamijo

**Affiliations:** 1 Research Institute for Clinical Oncology, Saitama Cancer Center, Saitama 362-0806, Japan; tkamijo@saitama-pho.jp; 2 Cancer Prevention Center, Chiba Cancer Center Research Institute, Chiba 260-8717, Japan; ynakamur@chiba-cc.jp; 3 Department of Childhood Cancer Data Management, National Center for Child Health and Development, Tokyo 157-8535, Japan; takimoto-t@ncchd.go.jp; 4 Department of Clinical Research, Saitama Children’s Medical Center, Saitama 330-8777, Japan; nkzw221@gmail.com; 5 Department of Pediatric Surgery, Chiba University Graduate School of Medicine, Chiba 260-8677, Japan; tomoro.hishiki@gmail.com; 6 Children’s Cancer Center, National Center for Child Health and Development, Tokyo 157-8535, Japan; matsumoto-kmk@ncchd.go.jp; 7 Department of Pediatrics, National Center for Global Health and Medicine, Tokyo 162-8655, Japan; hshichino@hosp.ncgm.go.jp; 8 Department of Pediatrics, Graduate School of Medical Science, Kyoto Prefectural University of Medicine, Kyoto 602-8566, Japan; iehara@koto.kpu-m.ac.jp; 9 Division of Cancer Genetics, Chiba Cancer Center Research Institute, Chiba 260-8717, Japan; nagasehiroki@hotmail.com; 10 Department of Pediatric Hematology and Oncology, Saitama Medical University International Medical Center, Saitama 350-1298, Japan; fksmtks1@saitama-med.ac.jp; 11 Department of Health Services Research, Institutes of Medicine, University of Tsukuba, Ibaraki 305-8576, Japan; 12 Division of Surgery, Surgical Oncology, National Center for Child Health and Development, Tokyo 157-8535, Japan; akihiroyo@gmail.com; 13 Division of Pediatric Surgical Oncology, National Cancer Center Hospital, Tokyo 104-0045, Japan; 14 Department of Pediatric Surgery, Faculty of Medical Sciences, Kyushu University, Fukuoka 812-8582, Japan; tajiri.tatsuro.909@m.kyushu-u.ac.jp; 15 Kyushu International Heavy Particle Beam Cancer Radiotherapy Center (SAGA HIMAT Foundation), Saga 841-0071, Japan; nakagawara-akira@saga-himat.jp

**Keywords:** neuroblastoma, genomic subgroup, prognostic factor, INRG, JCCG-JNBSG

## Abstract

Neuroblastomas (NBs) exhibit broad and divergent clinical behaviors and tumor risk classification at diagnosis is crucial for the selection of an appropriate therapeutic strategy for each patient. The present study aimed to validate the clinical relevance of International Neuroblastoma Risk Group (INRG) prognostic and genomic markers in a Japanese NB cohort using a retrospective analysis. Follow-up data based on 30 common INRG queries in 605 NB cases diagnosed in Japan between 1990 and 2014 were collected and the genome signature of each tumor sample was integrated. As previously indicated, age, tumor stage, *MYCN*, DNA ploidy, the adrenals as the primary tumor site, serum ferritin and lactate dehydrogenase (LDH) levels, segmental chromosome aberrations, and the number of chromosome breakpoints (BP) correlated with lower survival rates, while the thorax as the primary tumor site and numerical chromosome aberrations correlated with a favorable prognosis. In the patient group with stage 4, *MYCN* non-amplified tumors (n = 225), one of the challenging subsets for risk stratification, age ≥ 18 months, LDH ≥ 1400 U/L, and BP ≥ 7 correlated with lower overall and event-free survival rates (*p* < 0.05). The genome subgroup GG-P2s (partial chromosome gain/loss type with 1p/11q losses and 17q gain, n = 30) was strongly associated with a lower overall survival rate (5-year survival rate: 34%, *p* < 0.05). Therefore, the combination of the tumor genomic pattern (GG-P2s and BP ≥ 7) with age at diagnosis and LDH will be a promising predictor for *MYCN*-non-amplified high-risk NBs in patient subsets, in accordance with previous findings from the INRG project.

## 1. Introduction

Neuroblastomas (NBs), the most common extracranial solid cancer occurring in childhood, are characterized by broad and divergent clinical behaviors [1,2,3,4]. Tumors in infants are mostly favorable and often show spontaneous maturation or regression, whereas those in patients older than 18 months are more likely to grow aggressively and are often associated with a fatal outcome even with multimodality therapy. Risk stratification based on patient and tumor characteristics at diagnosis is vital for selecting the most appropriate treatment.

Multiple genome analyses have been conducted to characterize and classify tumor subsets with a heterogeneous clinical phenotype. The findings obtained revealed that NB subsets with a favorable prognosis generally possess the hyperdiploid karyotype of chromosomes, while other subsets with an unfavorable prognosis possess the diploid or tetraploid karyotype of chromosomes [5,6] and often have *MYCN* amplification [7], partial (segmental) deletions in chromosome arms 1p, 3p, 4p, and 11q, and partial gains in chromosome arms 1q, 2p, and 17q [8,9,10,11,12,13,14]. Array-based comparative genome hybridization (array CGH) revealed several types of global genomic aberrations in NBs to define the prognosis of patients [13,14]. In our previous study, a genome group (GG) of aberration silent (with no obvious losses and gains, except for *MYCN* amplification) [GG-S, 5-year cumulative survival rate (SR): 68%], that of partial chromosomal gains and/or losses (GG-P, SR: 43%), and that of whole chromosomal gains and/or losses (GG-W, SR: 80%) were defined in NBs [13,15]. The further subcategorization of these three groups was based on signatures with strong correlations with prognosis (1p loss, *MYCN* amplification, and 11q loss), resulting in several cohorts with highly contrasting outcomes. Similarly, Schleiermacher et al. reported that a higher number of chromosome breakpoints (BPs) correlated with an advanced stage of disease and a poorer outcome [16], and proposed a tumor subclassification using the structural alterations of ‘segmental chromosome aberration (SCA)’ and ‘numerical chromosome aberration (NCA)’ in addition to the *MYCN* amplification [14]. They indicated that tumors presenting exclusively with NCA (‘NCA only’) were associated with excellent survival, whereas the presence of SCA with or without *MYCN* amplification was a strong predictor of higher risk patients in low-and intermediate-risk NBs [14,17,18].

In 2005, an International Neuroblastoma Risk Group (INRG) Task Force was formed with representations from major pediatric cooperative groups around the world to establish a consensus approach for pretreatment risk stratification according to common criteria [19,20]. Risk criteria incorporated into the INRG classification system were based on statistical analyses of multiple prognostic factors in a cohort of 8800 patients whose data were provided by Australian, European, Japanese, and North American groups in the INRG Data Commons. The current INRG classification system was constructed in 2009 with seven potential prognostic factors, including tumor stage, histology, *MYCN* amplification, age at diagnosis, the 11q aberration, and DNA ploidy, to stratify patients into four risk categories: very low, low, intermediate, and high risk [19,21]. Although the INRG classification system provides a simple platform by applying easily accessible clinical markers, more recently identified genomic and molecular markers, including SCAs [14], copy number changes [15], individual gene mutations [4], gene expression profiles [22,23], and telomere maintenance mechanisms [24,25], have not yet been incorporated. In addition, the four risk categories defined by the current INRG markers still contain heterogeneous subsets, e.g., an ultra-high-risk subset, a subpopulation in high-risk NB patients with a particularly poor outcome [26]. The INRG and individual collaborative groups subsequently conducted statistical analyses of prognostic variables to predict ultra-high-risk NBs, indicating that elevated levels of serum lactate dehydrogenase (LDH) and ferritin and the pattern and burden of metastatic disease at diagnosis were strongly prognostic in all and within high-risk NB patients [27,28,29,30]. Further analyses to test the combination of existing and additional novel markers as well as an enrichment of the INRG Data Commons are needed in order to refine the risk classification.

We herein conducted a retrospective study on INRG prognostic markers as well as genomic markers in 605 Japanese patients with NB. The global genome signature of each tumor sample was also integrated into the analysis. The prognostic significance of the SCA and INRG markers in the whole cohort and that of the combination of tumor genomic patterns (GG-P2s and BP ≥ 7) with age at diagnosis and LDH in *MYCN* non-amplified, stage 4 NBs were confirmed. The present study increases the number of NB cases, mostly with Asian genetic backgrounds, to 1075 in the current INRG Data Commons.

## 2. Materials and Methods

### 2.1. Patient Cohort and Data Variables

Data were collected on patients registered in the Japan Childhood Cancer Group Neuroblastoma Committee (JCCG-JNBSG) database by the follow-up survey JNB-FU-2014. Between 1998 and 2008, we collected basic clinical and outcome data several times for NB cases registered by more than 130 hospitals throughout Japan to the Chiba Cancer Center for a molecular diagnosis. Most of the follow-up data were subsequently shared with JCCG-JNBSG, which was officially established in 2014. In the same year, to participate in the INRG collaboration for developing an interactive database for NB (iINRGdb), we expanded the collection items of clinical information according to 34 common metrics in the iINRGdb for the follow-up JNB-FU-2014. Eligibility for inclusion in the present study included (1) a confirmed diagnosis of NB, ganglioneuroblastoma (GNB), or ganglioneuroma (GN); (2) age no older than 21 years; (3) a known outcome; (4) DNA samples available for genomic analysis; and (5) informed consent. In addition to outcome data, the following clinical information on 12 risk factors was collected: age at diagnosis, INSS stage, *MYCN* status, DNA ploidy, serum ferritin and LDH levels at diagnosis, six primary tumor sites, eight metastatic sites, International Neuroblastoma Pathology Classification (INPC) or Shimada histological classification (favorable or unfavorable), diagnosis (NB, GNB, or GN), grade (differentiated or poorly/undifferentiated), and MKI (Low: <2% or <100 in 5000 cells, Intermediate: 2–4% or 100–200 in 5000 cells or High: >4% or >200 in 5000 cells). Fresh, frozen tumor specimens were obtained after informed consent from patients or guardians at hospitals belonging to the JNBSG study group. *MYCN* amplification was assessed by fluorescence in situ hybridization (FISH) and real-time quantitative PCR, and DNA ploidy was analyzed by flow cytometry [13]. All tumor samples were subjected to a histological review by a pathologist to confirm the diagnosis and assess the overall tumor content (>60%).

After diagnosis, patients with high-risk tumors were treated using intensive multi-modality therapy with stem cell or bone marrow transplants [31,32,33], while those with low- or intermediate-risk tumors were treated with conventional-dose chemotherapy plus surgery [34,35,36] or surgery alone.

### 2.2. Genomic Profile

To analyze 1p loss, 11q loss, and 17q gain, array CGH was performed using DNA prepared from tumor tissue obtained at diagnosis from 605 patients. A series of Agilent Human CGH Microarrays (44 B, 4 × 44 K, 8 × 60 K, 4 × 180 K, and 244 K, Agilent Technologies, Inc. Santa Clara, CA), Affymetrix GeneChip (250 K NspI, Affymetrix, Inc., Santa Clara, CA, USA), and custom UCSF Bacterial Artificial Chromosome (BAC) arrays [13] were used to analyze 465, 167, and 88 tumors, respectively, according to the manufacturers’ protocols. Among these, 162 Affymetrix data and 53 BAC data were previously reported (GSE12494 and GSE5784, https://www.ncbi.nlm.nih.gov/geo/ accessed on 26 October 2021) [13,37,38]. The number of BPs was counted according to the method of Schleiermacher et al. [16,17]. The chromosome aberration pattern was also elucidated based on the classification of NCA and SCA: NCA was defined as probe ratios homogeneously altered throughout the entire chromosome from the median copy number across the genome. SCA was defined by the presence of either at least three contiguous BAC or >3 Mb contiguous oligonucleotide probes exhibiting a genomic status different from that of the rest of the chromosome [17]. Cases representing only NCA were categorized as the genetic subtype “NCA only”, and those with SCA with or without NCA patterns as the genetic subtype “SCA”. The silent subtype with no obvious genetic changes was excluded from the survival analysis because of the difficulties associated with its interpretation. Another genomic classification (GG) was based on 17q gain/17 whole gain in combination with 1p loss, 11q loss, and *MYCN* amplification (see Table S1) [13,15].

### 2.3. Survival Analyses

Survival was estimated using the Kaplan–Meier method, and subgroups were compared using the Log-rank test. Overall survival (OS) and event-free survival (EFS) were expressed as a 5-year estimate ± the standard error (SE). Regarding OS, the time to an event was calculated as the time from diagnosis until death or the time of the last contact, if alive. EFS was defined as the time from diagnosis to the first episode of relapse, progression, second malignancy, or death. Patients who did not experience an event were censored at the time of the last follow-up. A Cox proportional hazards regression model was used to calculate the hazard ratio (HR) for an increased risk of an event (poor outcome category versus better outcome category).

In survival analyses, two binary variables were created. Regarding LDH and ferritin, 1400 U/L (indicated by Moreno et al.) [30] and 250 ng/mL (indicated by Morgenstern et al.) [28] were used to dichotomize the cohort as high or low, respectively. The number of BPs was divided into <7 and ≥7. The risk score proposed by Morgenstern et al. incorporating age (>5 years, 2 points), serum LDH (>1250 U/L, 1 point), and the number of metastatic systems (>1, 2 points) was also used in the survival analysis by comparisons of scores 1, 2, and 3 to score ≥ 4 [28].

All statistical calculations and data visualization were performed using JMP13.2.0 software (SAS Institute Inc., Cary, NC, USA). *p* values < 0.05 were considered to be significant.

## 3. Results

### 3.1. Patient Characteristics

As a cooperative effort by the Japan Neuroblastoma Study Group (JNBSG) in 2014 (JNB-FU-2014), follow-up data of 1985 NB patients were collected from 112 hospitals in Japan. Approximately 30 items were surveyed according to common INRG queries, including age at diagnosis, year of diagnosis, initial treatment, tumor stage, *MYCN* status, DNA ploidy, chromosomal aberrations in 1p, 11q, and 17q, serum ferritin and LDH levels, primary tumor sites (five and other sites), metastatic tumor sites (seven and other sites), pathological information, race, sex, site of relapse, secondary malignancy, and OS and EFS. In Japan, a nationwide mass screening of infants for NBs by urinary catecholamine metabolite levels was conducted between 1984 and 2003 [39]. Since an increased percentage

Since an increased percentage of infant NBs was observed during that period, we excluded mass screening-positive patients from the present study. A total of 605 patients met the eligibility criteria (JNB-FU-2014-605) of a confirmed diagnosis with the INSS stage, an age no older than 21 years, a known outcome, and DNA samples available for genomic analysis. The year of diagnosis of patients in the cohort was between 1990 and 2014, and the INSS stage distribution was 12% stage 1 (n = 73), 8% stage 2 (n = 48), 18% stage 3 (n = 107), 56% stage 4 (n = 341), and 6% stage 4s (n = 36). The median age at diagnosis was 24 months (range, 0 to 222 months), 146 (24%) tumors had *MYCN* amplification, and 187 out of 463 (40%) exhibited hyperdiploidy (Table 1). Patients were treated according to standard protocols in Japan [31,32,33,34,35,36] and no patients in this cohort received anti-GD2 immunotherapy. The median follow-up of patients without an event was 78 months (range: 0 to 233 months). Five-year OS and EFS rates in the patient cohort were 70 ± 2 and 53 ± 2.5%, respectively (Table 1).

### 3.2. Prognostic Significance of Markers in 605 Patients in All INSS Stages

#### 3.2.1. Prognostic Significance of Clinical Factors

The distribution of representative clinical characteristics is shown in Table 1. Regarding LDH and ferritin markers, we examined several cut-off values used by Moreno et al. (LDH: 1400 U/L; ferritin: 30 ng/mL) [30], by Morgenstern et al. (LDH: 1250 U/L; ferritin: 250 ng/mL) [28], and by the median values in this cohort (LDH: 740 U/L; ferritin: 140 ng/mL) to dichotomize patients for the survival analysis. Since LDH ≥ 1400 U/L and ferritin ≥ 250 ng/mL were identified as significant predictors of poor OS and EFS (Log-rank-*p* < 0.0001; both) in JNB-FU-2014-605, these cut-off values were used in subsequent analyses. Among primary and metastatic tumor sites, the adrenals as the primary site and metastasis to bone marrow, bone, distant lymph nodes, and the lungs correlated with a poor prognosis, while the thorax as the primary tumor site correlated with a good prognosis, which is consistent with previous findings [40] (Table 1). The INPC histological classification “unfavorable histology” and “high MKI” also correlated with poor OS and EFS. Metastasis to the liver and “NB or GNB nodular” were only significant for OS, not EFS.

#### 3.2.2. Assessment of the Risk Score by Morgenstern et al. as a High-Risk Marker

Morgenstern et al. proposed a “risk score” incorporating age (>5 years, 2 points), serum LDH (>1250 U/L, 1 point), and the number of metastatic systems (metastatic site index; MSI >1, 2 points) for the risk stratification of high-risk metastatic NB with a score of 5 points as an ultra-high-risk marker [28]. It can identify a patient subpopulation with 5-year EFS < 10% in the HR-NBL-1/SIOPEN study, but its validation in independent NB cohorts has not been reported yet. This risk score was available for 380 out of 605 JNB-FU-2014-605 patients, among which nine had a score = 5 (9/380, 2%). These nine patients consisted of eight stage 4 and one stage 3 cases, four *MYCN*-amplified, four deceased (OS: 22 to 57 months, EFS time: 15 to 27 months), two with an event (EFS: 16 and 84 months), and three censored with no event (survival time, 65 to 182 months). Since this subgroup was small, OS and EFS were compared between patients with scores of 1, 2, or 3 and ≥4. Unexpectedly, the patient subgroup with a score = 3 showed the worst 5-year OS and EFS rates (42 ± 6.0%, n = 76, and 26 ± 5.6%, n = 66, respectively) in JNB-FU-2014-605, while patients with a score ≥ 4 had a 5-year OS rate of 54 ± 8.2% (n = 44) and 5-year EFS rate of 39 ± 8.1% (n = 41) (Figure S1). When the cohort was dichotomized by a score of 1, 2, or 3 (n = 213) and ≥4 (n = 44), the two subgroups did not show a significant difference in OS or EFS (data not shown), possibly because age >5 years at diagnosis did not correlate with poor OS (*p* = 0.08) or EFS (*p* = 0.33) in JNB-FU-2014-605 patients.

#### 3.2.3. Prognostic Significance of Genomic Factors 1p, 11q, and 17q

To identify the genomic status of tumors, including 1p, 11q, 17q, and overall chromosomal aberration features, array CGH was conducted on 605 cases and the clinical relevance of each factor in JNB-FU-2014-605 was assessed. As expected, 1p loss, 11q loss, and 17q gain correlated with poor 5-year OS and EFS rates (Table 2, *p* < 0.0001). The strongest correlation among the three was 17q gain, with 5-year OS rates of 56 ± 3% (17q gain+, n = 340) vs. 87 ± 2.2% (no 17q gain, n = 265) and 5-year EFS rates of 37 ± 3.1% (17q gain+, n = 262) vs. 56 ± 3% (no 17q gain, n = 188).

#### 3.2.4. Prognostic Significance of SCA and Other Genome Subgroups

**Figure 1:**
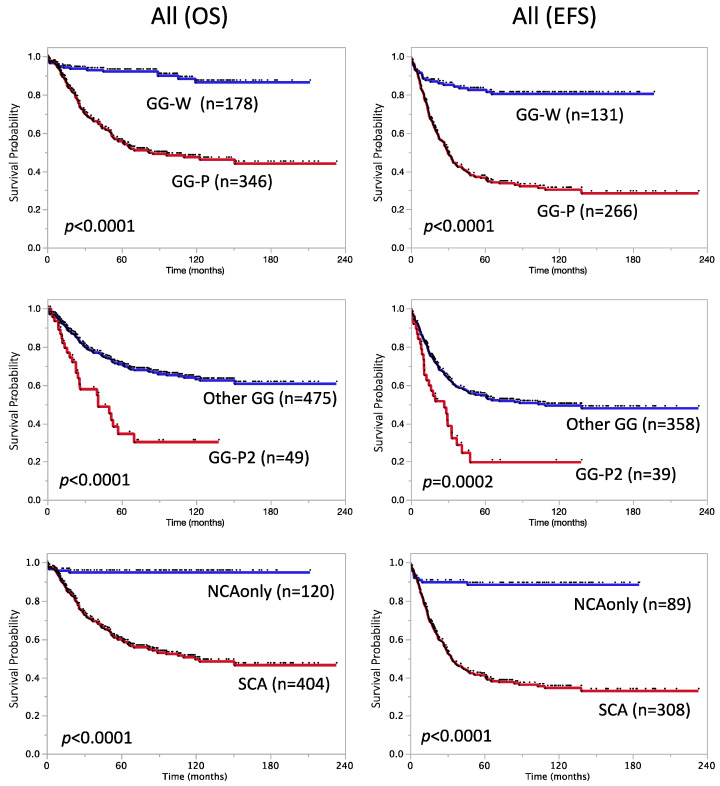
Kaplan–Meier survival curves of patients divided by genomic factors. OS (**left**) and EFS (**right**). OS: overall survival; EFS: event-free survival; GG: genome group; Other GG: GG-Wa, GG-Ws, GG-Pa (but no GG-P2a), or GG-Ps (but no GG-P2s); NCA: numerical chromosome aberration; SCA: segmental chromosome aberration.

As previously reported [13,15], tumors were classified into three GG according to array CGH results: silent (S), partial (P), and whole (W), which were further sub-grouped by *MYCN* amplification (a; *MYCN*-amp), 1p loss, 11q loss, and 17q gain (GG-P1-P5a/s, GG-W1-W5a/s, see Table S1). Concordant with our previous findings, GG-Ws (s: *MYCN* single copy) showed a favorable prognosis with 5-year OS and EFS rates of 93 ± 3.1% (n = 173) and 84 ± 3.4% (n = 126), respectively, whereas GG-Pa exhibited a poor prognosis (5-year OS rate: 48 ± 4.6%, n = 133, 5-year EFS rate: 34 ± 4.9%, n = 101) (Table S2). Overall, the GG-P signature (Pa + Ps) was associated with a significantly poorer prognosis than GG-W (Wa + Ws), with 5-year OS rates of 56 ± 2.9% (GG-P, n = 346) vs. 92 ± 2.1% (GG-W, n = 178) and 5-year EFS rates of 37 ± 3.1% (GG-P, n = 266) vs. 83 ± 3.4% (GG-W, n = 131) (*p* < 0.0001, Table 2 and Figure 1). Among GG subgroups, GG-Pa and GG-Ps with both 1p/11q losses (GG-P2a and GG-P2s, see Table S1) showed the most aggressive phenotypes (5-year OS rate: 34 ± 17%, n = 15, and 34 ± 9.7%, n = 34, respectively; 5-year EFS rate: 0 ± 0%, n = 11, and 23 ± 8.8%, n = 28, respectively, Table S2). Despite the small population, subgroup GG-P2 (P2a + P2s, namely with 1p loss, 11q loss, and 17q gain, but not with chromosome 17 whole gain) displayed a stronger correlation with poor OS and EFS than the other GG subgroups (*p* < 0.0001 and *p* = 0.0002, respectively, Table 2 and Figure 1).

**Table 2:** Genomic characteristics of 605 JPN patients (5-year overall and event-free survival rates).

Factor	N	5-Year OS ± SE (%)	Log-Rank-*p*	N	5-Year EFS ± SE (%)	Log-Rank-*p*
1p loss			<0.0001			<0.0001
Yes	192	50 ± 3.9		151	36 ± 4.1	
No	413	79 ± 2.2		299	62 ± 2.9	
11q loss			<0.0001			<0.0001
Yes	191	57 ± 4.1		146	36 ± 4.3	
No	414	75 ± 2.2		304	61 ± 2.9	
17q gain			<0.0001			<0.0001
Yes	340	56 ± 3.0		262	37 ± 3.1	
No	265	87 ± 2.2		188	76 ± 3.2	
Genome subgroup			<0.0001			<0.0001
GG-P (Pa + Ps)	346	56 ± 2.9		266	37 ± 3.1	
GG-W (Wa + Ws)	178	92 ± 2.1		131	83 ± 3.4	
GG-P2 subgroup			<0.0001			0.0002
GG-P2 (P2a + P2s)	49	34 ± 8.4		39	20 ± 7.6	
Other GG *	475	71 ± 2.3		358	55 ± 2.7	
Genetic subtype			<0.0001			<0.0001
NCA only	120	95 ± 2.0		89	89 ± 3.4	
SCA (typSCA+atypSCA)	404	60 ± 2.7		308	41 ± 2.9	
Breakpoints			<0.0001			<0.0001
<7	388	79 ± 2.2		271	67 ± 2.9	
≥7	217	53 ± 3.8		179	32 ± 3.7	

OS: overall survival; EFS: event-free survival; SE: standard error; GG: genome group; * Other GG: GG-Wa, GG-Ws, GG-Pa (but no GG-P2a), and GG-Ps (but no GG-P2s); NCA: numerical chromosome aberration; SCA: segmental chromosome aberration; typ: typical; atyp: atypical.

We then assessed the clinical significance of another genomic signature “SCA” being applied for the therapy stratification of intermediate- and low-risk NBs by the SIOPEN group [17,18]. After excluding 81 cases with GG-Ss (n = 73) and GG-Sa (n = 8) that were difficult to interpret, 524 out of 605 cases were classified into “SCA” or “NCA only” according to the definition by Schleiermacher et al. As previously reported [17,18], the survival curves of patients with the “SCA” and “NCA only” signatures were significantly separated (5-year OS rates: 60 ± 2.7%, n = 404 vs. 95 ± 2%, n = 120, *p* < 0.0001; 5-year EFS rates: 41 ± 2.9%, n = 308 vs. 89 ± 3.4%, n = 89, *p* < 0.0001) in JNB-FU-2014-605 patients (Table 2 and Figure 1), confirming the good potential of the SCA signature as a risk classification marker for NBs. We also compared patient survival with typical SCA (with 1p/3p/4p/11q losses or 1q/2p/17q gains, n = 392) and atypical SCA (any other SCA, except those defined as typSCA, n = 12), but did not find any significant difference (data not shown).

#### 3.2.5. Prognostic Significance of the Number of BPs

**Figure 2:**
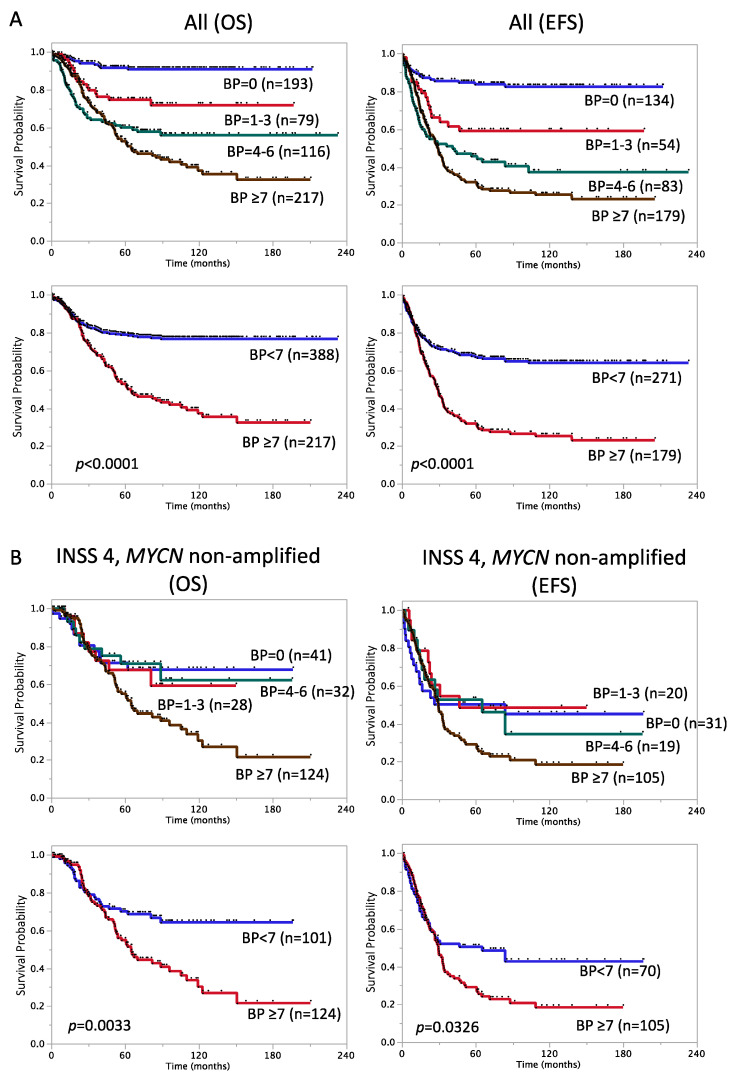
Kaplan–Meier survival curves of NB patients divided by the number of breakpoints. (**A**) OS and EFS of all patients. (**B**) OS and EFS of 225 stage 4, *MYCN* non-amplified cases. OS: overall survival; EFS: event-free survival; BP: the number of breakpoints.

In addition to the prognostic impact of SCA genetic features, Schleiermacher et al. reported that a higher number of chromosome BPs correlated with an advanced stage of disease and a poorer outcome [16]. To validate these findings in JNB-FU-2014-605, we counted the number of BPs in each sample using the method described by Schleiermacher et al. The number of BPs per tumor ranged between 0 (=NCA only) and 56 (median: 4). We confirmed that patient groups harboring a higher number of BPs in tumors had significantly lower SR in JNB-FU-2014-605 (Figure 2A). Five-year OS and EFS rates in the patient groups with BP < 7 vs. BP ≥ 7 were 79 ± 2.2% (n = 388) vs. 53 ± 3.8% (n = 217) and 67 ± 2.9% (n = 271) vs. 32 ± 3.7% (n = 179), respectively (*p* < 0.0001, Figure 2A). As expected, a higher number of BP was observed in GG-P compared to GG-W, and so was in GG-Ps compared to GG-Pa (BP < 7 vs. BP ≥ 7, Chi-square test, *p* < 0.001).

### 3.3. Factors with Prognostic Significance in Stage 4, MYCN Non-Amplified Cases

The patient group with stage 4, *MYCN* non-amplified tumors has been one of the challenging subsets for risk stratification [1,4]. A total of 225 out of 605 patients corresponded to this subset (5-year OS and EFS rates were 62 ± 3.6% and 38 ± 3.9%, respectively) and clinical and genomic markers, which showed the prognostic impact in all tumor stages described in Table 1 and Table 2, were verified for OS and EFS in a univariate analysis.

**Figure 3:**
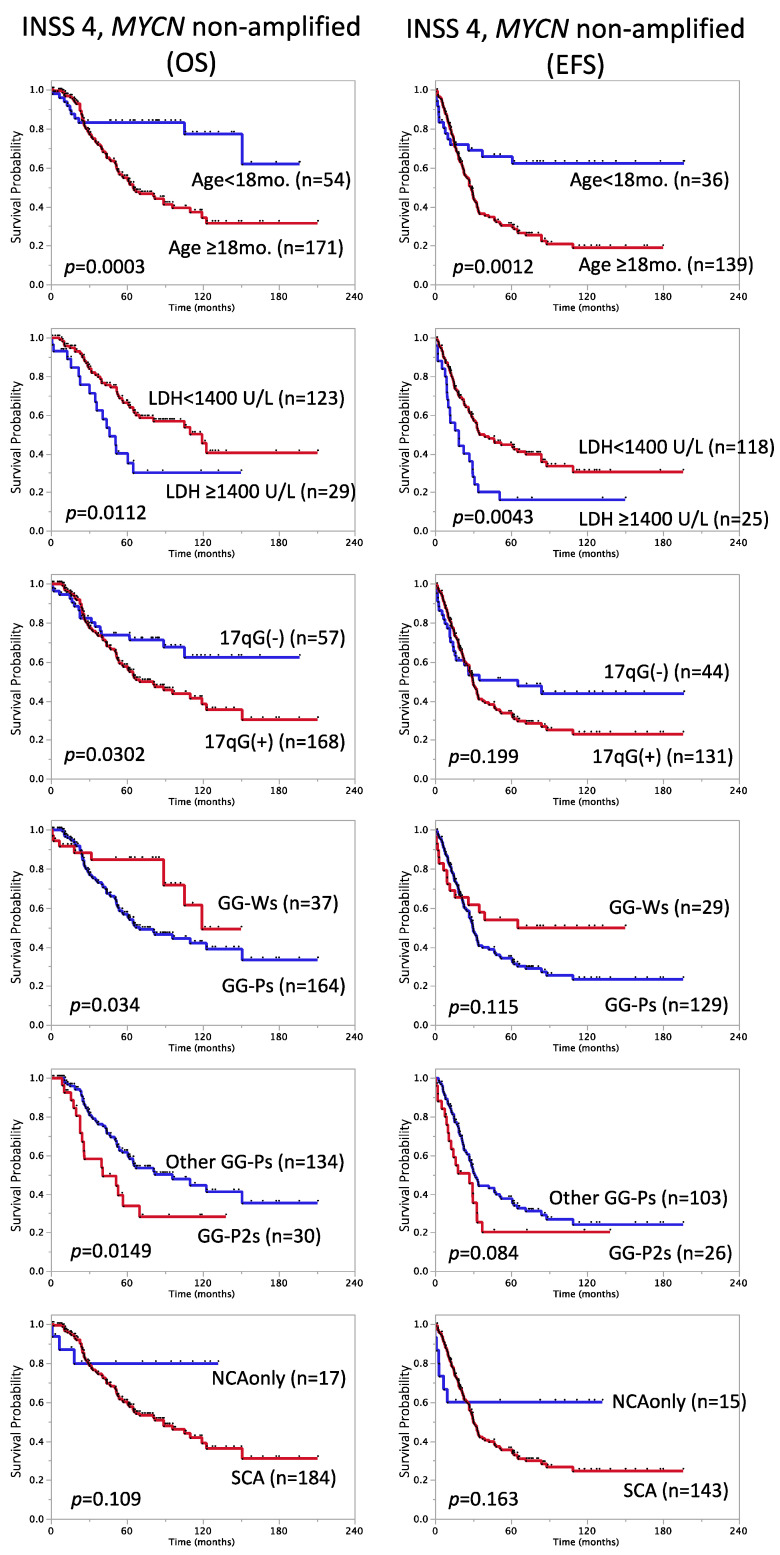
Kaplan–Meier survival curves of patients with stage 4, *MYCN* non-amplified tumors divided by clinical and genomic factors. OS (**left**) and EFS (**right**). OS: overall survival; EFS: event-free survival; mo.: months; GG: genome group; Other GG-Ps: GG-Ps (but no GG-P2s); NCA: numerical chromosome aberration; SCA: segmental chromosome aberration.

As shown in Table 3, Figure 2B and Figure 3, age ≥ 18 months [Cox HR: 3.2, 95% confidence interval (CI): 1.7–6.5], LDH ≥ 1400 U/L (HR: 2.1, 95% CI: 1.1–3.6), 17q gain (HR: 1.8, 95% CI: 1.1–3.2), GG-Ps (HR: 2.1, 95% CI: 1.1–4.5), GG-P2s (HR: 2.0, 95% CI: 1.1–3.4), and BP ≥ 7 (HR: 1.9, 95% CI: 1.2–3.1) showed correlations with lower SR in the patient subset with stage 4, *MYCN* non-amplified tumors (Log-rank-*p* < 0.05), while diploidy, ferritin ≥ 250 ng/mL, and SCA did not. Regarding 5-year EFS, age ≥ 18 months (HR: 2.5, 95% CI: 1.5–4.8), LDH ≥ 1400 U/L (HR: 2.0, 95% CI: 1.2–3.2), and BP ≥ 7 (HR: 1.5, 95% CI: 1.0–2.3) were still significant (Log-rank-*p* < 0.05), while ferritin ≥ 250 ng/mL (HR: 1.6, 95% CI: 1.0–2.6, *p* = 0.047) and GG-P2s (HR: 1.6, 95% CI: 0.9–2.6, *p* = 0.084) were associated with EFS (Table 3, Figure 2B and Figure 3). We also assessed the risk score ≥ 4 reported by Morgenstern et al. in this subset but did not find a significant relationship (data not shown). Therefore, in accordance with previous findings on high-risk NB patients [28,30], age ≥ 18 months and LDH ≥ 1400 U/L were confirmed as representative strong high-risk markers in stage 4, *MYCN* non-amplified cases.

### 3.4. Multivariable Survival Analysis of Stage 4, MYCN Non-Amplified Cases

We investigated whether the significance of each factor (significant in the univariate analysis of OS and EFS in Table 3) was retained in combination with age (≥18 months) or LDH (≥1400 U/L) within the subgroup of stage 4, *MYCN*-non-amplified cases in a multivariable Cox model. A bivariate analysis of OS in this subgroup indicated that only GG-P2s and LDH retained independent significance in combination with age (age: HR: 2.5, 95% CI: 1.1–7.10, *p* < 0.05; GG-P2s: HR: 1.9, 95% CI: 1.1–3.3, *p* < 0.05, and age: HR: 1.9, 95% CI: 0.9–4.3, *p* = 0.079; LDH: HR: 1.9, 95% CI: 1.1–3.4, *p* < 0.05), whereas only BP ≥ 7 retained significance with LDH (LDH: HR: 2.2, 95% CI: 1.2–3.8, *p* < 0.05; BP ≥ 7: HR: 2.0, 95% CI: 1.2–3.6, *p* < 0.05, Table 4). GG-P2s also showed a weaker relationship than BP ≥ 7 in combination with LDH for OS (LDH: HR: 2.1, 95% CI: 1.0–4.0, *p* = 0.054; GG-P2s: HR: 2.1, 95% CI: 1.0–3.9, *p* = 0.052). Similarly, each factor was tested by a bivariate analysis for EFS, and age with LDH, and LDH with BP ≥ 7 were independently associated with EFS (Table 4). In a trivariate analysis, LDH and BP ≥ 7, and age and LDH retained significance for OS and EFS, respectively. Therefore, the variables age ≥ 18 months, LDH ≥ 1400 U/L, and the genomic pattern (GG-P2s and BPs ≥ 7) will be good candidates for predicting the outcomes of patients with stage 4, *MYCN* non-amplified NB tumors.

## 4. Discussion

The present study provides basic clinical and genomic data on 605 Japanese NB patients to the INRG Data Commons. The clinical relevance of INRG risk factors, as well as genomic signatures, were confirmed in the Japanese cohort (1990–2014). Since many cases in this cohort were registered before the concept of the INRG staging system (INRGSS) by Monclair et al. [41], the INSS stage was used for the analysis. The SR of patients in each INSS stage was similar to the INRG analytic cohort reported by Ambros et al. [21], except for the stage 4s population having worse SR in our cohort (5-year OS rate: 75 ± 7.2%, n = 36; 5-year EFS rate: 74 ± 8.6%, n = 27). The overall SR of stage 4s patients diagnosed in 2003–2014 was 89 ± 7.4%, which was similar to the INRG cohort [21], whereas that in 1990–2002 was 61 ± 11%, suggesting that stage 4s patients with poor outcomes diagnosed before 2002 were unexpectedly accumulated in JNB-FU-2014-605.

As previously reported by the INRG consortium [19,27], the clinical markers of stage 4, age ≥ 18 months, *MYCN* amplification, diploidy, high levels of ferritin and LDH, the adrenals as the primary tumor site, metastasis to bone marrow, bone, distant lymph nodes, and the lungs, unfavorable histology, and high MKI correlated with poor OS and EFS in JNB-FU-2014-605. Metastasis to the liver and INPC NB or GNB nodular were also significant prognostic factors for poor OS, but less significant for EFS. Patients with thoracic primary tumors had better SR than those with non-thoracic tumors, as previously reported [40]. Since the basal levels of biomarkers may differ by race and ethnicity, we examined several reported cut-off values for LDH and ferritin levels in survival analysis and showed the potential of LDH ≥ 1400 U/L and ferritin ≥ 250 ng/mL as poor prognosis markers in our cohort.

The classification of high-risk and ultra-high-risk patients is needed for a precision therapeutic strategy for NB. We confirmed that MSI > 1 and LDH strongly correlated with a poor prognosis in Japanese patients; however, in contrast to previous findings [28], age > 5 years had no significant clinical impact in high-risk patients in our cohort (age ≥ 5 years vs. age < 5 years in stage 4, age > 1.5 years patients; *p* = 0.406 and *p* = 0.404 for OS and EFS, respectively). More recently, Moreno et al. constructed a nomogram of clinical and biological factors for the risk classification of high-risk NBs using *MYCN*, LDH, and metastasis to bone marrow [30]. In their analysis, age ≥ 5 years at diagnosis was significant, whereas its HR from univariate Cox models of OS for 1820 cases (≥18 months old with metastatic NBs, diagnosed in 1998–2015, in INRG Data Commons) was close to 1 [30]. Therefore, the clinical impact of age > 5 years appears to be smaller than other significant factors and may vary among patients with different genetic backgrounds.

In addition to clinical factors, such as LDH and MSI, genomic aberration features were also verified in the Japanese cohort. In JNB-FU-2014-605, the signature “NCA only” showed a good correlation with a favorable prognosis in patients (Figure 1), and the HR of the SCA marker was the highest among the genomic features examined (HR: 11, 95% CI: 5.1–27, *p* < 0.0001 for OS, HR: 7.5, 95% CI: 4.2–15, *p* < 0.0001 for EFS). Previous studies suggested that the SCA exhibits good potential for the stratification of non-high-risk NBs [17,18], and additional genomic markers are required for high-risk NBs. Our classification GG-P1~5 will be one of the candidates to combine with SCA; 17q partial gain was the best poor survival marker for all and stage 4, *MYCN* non-amplified patients among the typical chromosome aberrations, including 1p loss and 11q loss, which led us to its usage for the GG-P/GG-W classification. The GG-Ws subgroup is nearly equal to NCA and also showed good SR in all 605 cases (5-year OS rate: 93 ± 1.9%, n = 173; 5-year EFS rate: 84 ± 3.4%, n = 126), while GG-P2/GG-P2s, albeit a minor population, correlated with a poor prognosis for OS in stage 4, *MYCN*-non-amplified cases (Figure 3, Figure S2 and Figure S3, Table 3 and Table 4). This result implies that the greater accumulation of chromosomal aberrations is associated with the acquisition of a more aggressive phenotype in tumors. Of note, a clear association was observed between the number of BPs and patient prognosis in all 605 cases and in the stage 4, *MYCN*-non-amplified subset (Figure 2). Although it may currently be difficult to analyze the number of BPs for all tumor samples due to the high running cost, it is worth considering the adoption of this marker in future risk classifications.

Some limitations of the present study are the incomplete collection of some data, such as EFS, metastatic and relapse site information, and histology, and an analysis that was restricted to patients diagnosed between 1990 and 2014, before the availability of INRGSS and anti-GD2 immunotherapy [4,20]. EFS data were provided for 450 out of 605 registered cases and the follow-up time for 33 censored cases was shorter than 24 months. Metastatic and relapse sites were according to the information entered by individual hospitals, not by a central review. These incomplete items may be filled in the future by our continuous follow-up efforts. A web-based, interactive system for case registration and data collection was established in 2016 by the JCCG-JNBSG data center, which made the database more integrative and sustainable, particularly for recently registered patients. Another limitation in the present study is that only global genomic alterations were used as genomic markers. In addition to genomic subgrouping and the number of BPs, specific tumor genetic aberrations, including *ALK* mutation/amplification [37,42], abnormal telomere maintenance mechanisms (*TERT* rearrangements, the alternative lengthening of telomeres, and high *TERT* expression) [24,25], *ATRX* alterations [43], and mutations in TP53/RAS/MAPK signaling pathway-related genes [24,44,45] have been shown to correlate with patient prognosis. This genomic information is being collected, particularly on patients registered in JCCG-JNBSG clinical studies and will be integrated into comprehensive analyses of multiple genomic factors by the INRG genomics committee for consideration in the next version of the INRG risk classification.

In summary, the present study provides 605 Japanese NB data with clinical and genomic factors in the INRGdb. The prognostic significance of current INRG risk factors was confirmed in the cohort, but age ≥ 5 years as a high risk factor needs to be further assessed in Japanese NBs. Serum LDH (≥1400 U/L), age ≥ 18 months, BP ≥ 7 and genome subgroup GG-P2s will be good candidates to be included in the next version of the risk score to identify particularly high risk NB populations in *MYCN*-non-amplified stage 4 patients. Accumulating multi-omics data from the clinical studies by international collaborative efforts will accelerate the establishment of more precise pretreatment risk stratification for high-risk NBs.

## Figures and Tables

**Table 1:** Clinical characteristics of 605 JPN patients (5-year overall and event-free survival rates).

Factor	N	5-Year OS ± SE (%)	Log-Rank-*p*	N	5-Year EFS ± SE (%)	Log-Rank-*p*
Overall cohort	605	70 ± 2.0		450	53 ± 2.5	
INSS stage			<0.0001			<0.0001
stage 1,2,3,4s	264	89 ± 2.0		185	79 ± 3.1	
stage 4	341	54 ± 3.0		265	35 ± 3.1	
Age at diagnosis			<0.0001			<0.0001
<18 months	254	81 ± 2.5		172	69 ± 3.6	
≥18 months	351	62 ± 2.8		278	43 ± 3.1	
*MYCN* amplification			<0.0001			<0.0001
Not amplified (<10 copies)	459	77 ± 2.2		337	60 ± 2.8	
Amplified (≥10 copies)	146	47 ± 4.4		113	34 ± 4.6	
DNA ploidy			<0.0001			<0.0001
Hyperdiploidy	187	87 ± 2.7		138	75 ± 3.9	
Diploidy	276	62 ± 3.2		196	43 ± 3.7	
Ferritin			0.0003			<0.0001
<250 ng/mL	215	75 ± 3.2		195	62 ± 3.6	
≥250 ng/mL	94	56 ± 5.8		87	40 ± 5.7	
LDH			<0.0001			<0.0001
<1400 U/L	288	79 ± 2.6		274	65 ± 3.0	
≥1400 U/L	109	43 ± 5.2		96	29 ± 4.8	
Primary site of tumor						
Adrenal			0.0003			0.0032
No	237	78 ± 2.9		174	63 ± 3.8	
Yes	363	65 ± 2.7		273	47 ± 3.2	
Thorax			0.0002			0.0017
No	531	67 ± 2.2		398	50 ± 2.6	
Yes	69	90 ± 3.8		49	78 ± 6.1	
Metastatic site (MET)						
MET_Bone marrow			<0.0001			<0.0001
No	231	81 ± 2.7		218	72 ± 3.2	
Yes	203	53 ± 3.9		188	34 ± 3.7	
MET_Bone			<0.0001			<0.0001
No	233	82 ± 2.7		221	71 ± 3.2	
Yes	196	53 ± 3.9		181	35 ± 3.7	
MET_DLN			0.0002			<0.0001
No	274	75± 2.8		262	63 ± 3.1	
Yes	148	58 ± 4.4		134	39 ± 4.4	
MET_Liver			0.0151			0.0653
No	343	70 ± 2.7		320	55 ± 2.9	
Yes	90	60 ± 5.4		83	50 ± 5.6	
MET_Lung			0.0368			0.0314
No	416	69 ± 2.4		388	55 ± 2.6	
Yes	16	44 ± 13.3		15	33 ± 12.2	
Histological classification (INPC)			<0.0001			<0.0001
Favorable	131	84 ± 3.3		122	78 ± 3.9	
Unfavorable	189	59 ± 4.0		177	40 ± 3.9	
Diagnosis (INPC)			0.0188			0.0872
NB, GNB nodular	316	68 ± 2.9		296	53 ± 3.1	
Others *	28	92 ± 5.3		24	79 ± 8.6	
MKI (INPC)			0.0015			0.0034
Low or Intermediate	173	77 ± 3.6		164	63 ± 4.0	
High	54	57 ± 7.4		51	38 ± 7.5	

OS: overall survival; EFS: event-free survival; SE: standard error; INSS: International Neuroblastoma Staging System; LDH: lactate dehydrogenase; DLN: distant lymph node; INPC: International Neuroblastoma Pathology Classification; * Diagnosis “Others”: GNB intermixed (Schwannian stroma-rich), GN (Schwannian stroma-dominant) maturing/mature subtype or GN; MKI: Mitosis-karyorrhexis index.

**Table 3:** Prognostic significance of individual factors in stage 4, *MYCN* non-amplified cases.

Factor	N	5-Year OS ± SE (%)	Log-Rank-*p*	HR	(95% CI)	N	5-Year EFS ± SE (%)	Log-Rank-*p*	HR	(95% CI)
Stage 4, *MYCN* non-amplified	225	62 ± 3.6				175	38 ± 3.9			
Age at diagnosis			0.0003	3.2	1.7–6.5			0.0012	2.5	1.5–4.8
<18 months *	54	83 ± 5.4				36	66 ± 8.1			
≥18 months	171	55 ± 4.3				139	30 ± 4.2			
DNA ploidy			0.545	1.2	0.7–2.2			0.188	1.4	0.9–2.4
Hyperdiploidy *	47	71 ± 7.4				40	45 ± 8.7			
Diploidy	117	61 ± 5.1				87	39 ± 5.5			
Ferritin			0.128	1.6	0.9–2.9			0.0471	1.6	1.0–2.6
<250 ng/mL *	70	68 ± 6.2				64	47 ± 6.5			
≥250 ng/mL	55	57 ± 8.1				51	33 ± 7.5			
LDH			0.0112	2.1	1.1–3.6			0.0043	2.0	1.2–3.2
<1400 U/L *	123	66 ± 4.8				118	45 ± 4.9			
≥1400 U/L	29	40 ± 10.3				25	16 ± 7.3			
1p loss			0.07	1.5	0.9–2.4			0.203	1.3	0.8–2.0
Yes	49	51 ± 8.0				43	29 ± 7.4			
No *	176	65 ± 4.0				132	41 ± 4.5			
11q loss			0.0617	1.5	1.0–2.5			0.531	1.1	0.8–1.7
Yes	141	57 ± 4.7				110	35 ± 4.9			
No *	84	70 ± 5.5				65	42 ± 6.3			
17q gain			0.0302	1.8	1.1–3.2			0.199	1.4	0.9–2.2
Yes	168	58 ± 4.3				131	34 ± 4.4			
No *	57	74 ± 6.3				44	51 ± 7.8			
Genome Group			0.034	2.1	1.1–4.5			0.115	1.6	0.9–2.9
GG-Ps	164	57 ± 4.4				129	34 ± 4.4			
GG-Ws *	37	85 ± 6.3				29	54 ± 9.5			
GG-P2s subgroup			0.0149	2.0	1.1–3.4			0.084	1.6	0.9–2.6
GG-P2s	30	34 ± 10.3				26	20 ± 8.8			
Other GG *	134	62 ± 4.7				103	38 ± 5.0			
Genetic subtype			0.109	2.5	0.9–10.2			0.163	1.8	0.9–4.6
NCA only *	17	80 ± 10.5				15	60 ± 12.7			
SCA (typSCA+atypSCA)	184	60 ± 4.1				143	36 ± 4.2			
Breakpoints			0.0033	1.9	1.2–3.1			0.0326	1.5	1.0–2.3
<7 *	101	70 ± 5.0				70	50 ± 6.2			
≥7	124	55 ± 5.1				105	29 ± 4.8			

A Cox proportional hazards regression model was used to calculate the hazard ratio for an increased risk of an event (poor outcome category versus better outcome category). *: better outcome category. OS: overall survival; EFS: event-free survival; SE: standard error; HR: hazard ratio; CI: confidence interval; LDH: lactate dehydrogenase; GG: genome group; Other GG: GG-Ws or GG-Ps (but no GG-P2s); NCA: numerical chromosome aberration; SCA: segmental chromosome aberration; typ: typical; atyp: atypical.

**Table 4:** Multivariate analysis of OS and EFS according to combinations of variables in stage 4, *MYCN* non-amplified patients.

Combination of Variables	OS				EFS			
	N	HR	(95% CI)	*p*-Value	N	HR	(95% CI)	*p*-Value
Age ≥ 18 months	152	1.9	0.9–4.3	0.0789	143	2.4	1.3–5.0	0.004
LDH ≥ 1400 U/L		1.9	1.1–3.4	0.0323		1.9	1.1–3.0	0.0194
Age ≥ 18 months	164	2.5	1.1–7.1	0.0291	129	2.0	0.9–5.1	0.0799
GG-P2s subgroup		1.9	1.1–3.3	0.0325		1.6	0.9–2.7	0.0838
Age ≥ 18 months	225	2.7	1.4–5.6	0.0023	175	2.3	1.3–4.5	0.0035
Breakpoints ≥ 7		1.5	0.9–2.4	0.0919		1.2	0.8–1.9	0.3361
LDH ≥ 1400 U/L	152	2.2	1.2–3.8	0.0138	143	2.1	1.3–3.4	0.006
Breakpoints ≥ 7		2.0	1.2–3.6	0.0098		1.7	1.1–2.7	0.0181
Age ≥ 18 months	152	1.4	0.6–3.3	0.4604	143	2.0	1.0–4.4	0.0396
LDH ≥ 1400 U/L		2.1	1.1–3.7	0.0184		2.0	1.2–3.2	0.0131
Breakpoints ≥ 7		1.8	1.0–3.5	0.0422		1.4	0.8–2.3	0.2123

OS: overall survival; EFS: event-free survival; HR: hazard ratio; CI: confidence interval; LDH: lactate dehydrogenase; GG: genome group.

## Data Availability

Clinical data and genomic features (SCA/NCA, GG-P/W/S, and the number of BPs) used in this study are available at http://inrgdb.org/publication-policy/apply/(INRGDC) or upon request. Array CGH data (162 Affymetrix data and 53 BAC data) were previously reported (GSE12494 and GSE5784, https://www.ncbi.nlm.nih.gov/geo/).

## Supplementary Materials

The following supporting information can be downloaded at: www.mdpi.com/article/10.3390/biom12010018/s1, Figure S1: Prognostic significance of the risk score reported by Morgenstern et al., Figure S2: Frequency of 1p loss, 11q loss, or 17q gain in 183 “segmental” subtypes detected in stage 4, *MYCN* non-amplified cases, Figure S3: Frequency of 1p loss, 11q loss, or 17q gain in 265 ”segmental” subtypes detected in all stage 4, *MYCN* non-amplified cases, Table S1: Characteristic features of each genomic subgroup with its detected number in 605 JPN cases, Table S2: Five-year overall and event-free survival rates in genomic subgroups.
