# Supplementary material for: Retrospective Analysis of INRG Clinical and Genomic Factors for 605 Neuroblastomas in Japan: A Report from the Japan Children’s Cancer Group Neuroblastoma Committee (JCCG-JNBSG)
Source: Biomolecules. 2021 Dec 23;12(1):18. doi: 10.3390/biom12010018 (PMC8774029; doi:10.3390/biom12010018)
Supplement: Supplementary file 1 [file biomolecules-12-00018-s001.zip › biomolecules-1457545-SM-update.pdf]

Table S1. Characteristic feature of each genomic subgroup with its detected numbers in 605 JPN samples.

Genome subgroup (GG)	N	*MYCN* -amp	17WG	17qG	1pL	11qL	% of total cohort	SCA	NCA only
GG-Pa	133								
GG-P1a	101	+	-	+	+	-	major (17%)	101	-
**GG-P2a**	15	+	-	**+**	**+**	**+**	2.5%	15	-
GG-P3a	4	+	-	+	-	+		4	-
GG-P4a	3	+	-	+	-	-		3	-
GG-P5a	10	+	-	-	any	any		10	-
GG-Ps	213								
GG-P1s	20	-	-	+	+	-		20	-
**GG-P2s**	34	-	-	**+**	**+**	**+**	5.6%	34	-
GG-P3s	124	-	-	+	-	+	major (21%)	124	-
GG-P4s	27	-	-	+	-	-		27	-
GG-P5s	8	-	-	-	any	any		8	-
GG-Wa	5						rare		
GG-W1a	2	+	+	-	+	-		2	-
GG-W2a	0	+	+	-	+	+		0	-
GG-W3a	0	+	+	-	-	+		0	-
GG-W4a	3	+	+	-	-	-		3	-
GG-W5a	0	+	-	-	any	any		0	-
GG-Ws	173								
GG-W1s	5	-	+	-	+	-		5	-
GG-W2s	1	-	+	-	+	+		1	-
GG-W3s	11	-	+	-	-	+		11	-
GG-W4s	151	-	+	-	-	-	major (25%)	**34**	**117**
GG-W5s	5	-	-	-	any	any		**2**	**3**
GG-Sa	8	+	-	-	-	-	silent	-	-
GG-Ss	73	-	-	-	-	-	silent	-	-

GG: genome group; amp: amplification; 17WG: chromosome 17 whole gain (both short and long chromosomal arms); 17qG: chromosome 17q gain; 1pL: chromosome 1p loss; 11qL: chromosome 11q loss; SCA: segmental chromosome aberration; NCA: numerical chromosome aberration.

Table S2. Five-year overall and event-free survival rates of genomic subgroup.

Factor	N	5-year OS ± SE (%)	Log-rank-*p*	N	5-year EFS ± SE (%)	Log-rank-*p*
Genome Group						
GG-Pa	133	48 ± 4.6		101	34 ± 4.9	
GG-Ps	213	61 ± 3.8		165	38 ± 4.0	
GG-Wa	5	53 ± 24.8		5	53 ± 24.8	
GG-Ws	173	93 ± 1.9		126	84 ± 3.4	
GG-Pa subgroup						
GG-P1a	101	46 ± 5.3		75	35 ± 5.7	
GG-P2a	15	34 ± 17.3		11	0 ± 0.0	
GG-P3a	4	75 ± 21.7		4	25 ± 21.7	
GG-P4a	3	67 ± 27.2		2	50 ± 35.4	
GG-P5a	10	60 ± 15.5		9	44 ± 16.6	
GG-Ps subgroup						
GG-P1s	20	79 ± 9.5		19	42 ± 11.3	
GG-P2s	34	34 ± 9.7		28	23 ± 8.8	
GG-P3s	124	61 ± 5.0		93	38 ± 5.3	
GG-P4s	27	64 ± 10.3		22	42 ± 11.0	
GG-P5s	8	88 ± 11.7		3	100 ± 0.0	
GG-P2s subgroup			0.001			0.0482
GG-P2s	34	34 ± 9.7		28	23 ± 8.8	
Other GG*	179	65 ± 4.0		137	41 ± 4.4	
Genome Group			<0.0001			<0.0001
GG-Ps	213	61 ± 3.8		165	38 ± 4.0	
GG-Ws	173	93 ± 1.9		126	84 ± 3.4	

OS: overall survival; EFS: event-free survival; SE: standard error; GG: genome group; *Other GG: GG-Wa, GG-Ws, GG-Pa (but no GG-P2a), or GG-Ps (but no GG-P2s).

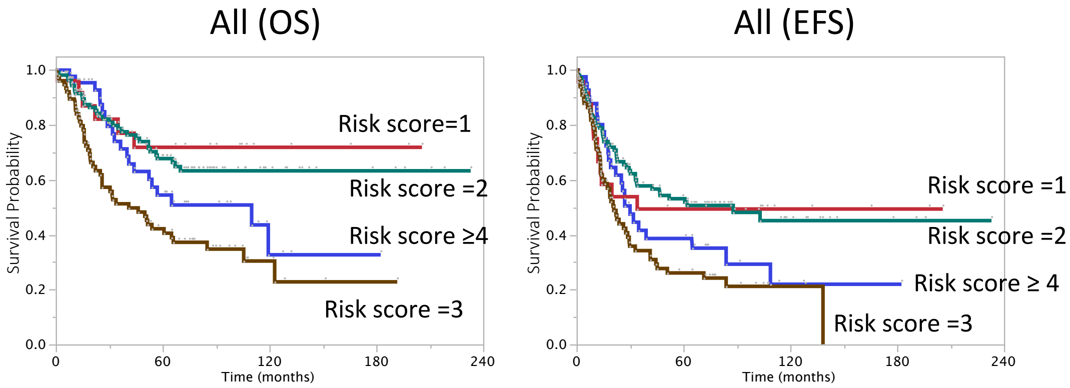

Factor	N	5-year OS	±	SE (%)	Log-rank-*p*	N	5-year EFS	±	SE (%)	Log-rank-*p*
Risk score (Morgenstern *et al.*)					<0.0001					0.0034
1	26	72	±	9.9		24	49	±	10.3	
2	111	68	±	4.9		106	53	±	5.1	
3	76	42	±	6.0		66	26	±	5.6	
≥4	44	54	±	8.2		41	39	±	8.1	

Figure S1. Prognostic significance of the risk score reported by Morgenstern *et. al.*

OS: overall survival; EFS: event-free survival; SE: standard error; MSI: metastatic site index

Genetic subtype "segmental (SCA)" detected in
Stage 4, *MYCN* non-amplified cases (n=184)

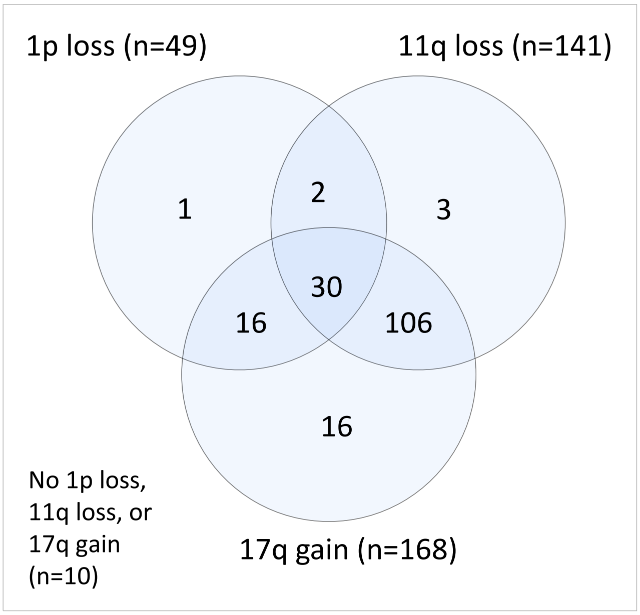

GG-P2 subtype (n=30) is accompanying 1pL,11qL, and 17qG.

Figure S2. Frequency of 1p loss, 11q loss, or 17q gain in 184 "segmental" subtypes detected in stage 4, *MYCN* non-amplified cases.

Genetic subtype "segmental (SCA) " detected in
MYCN non-amplified cases (n=266)

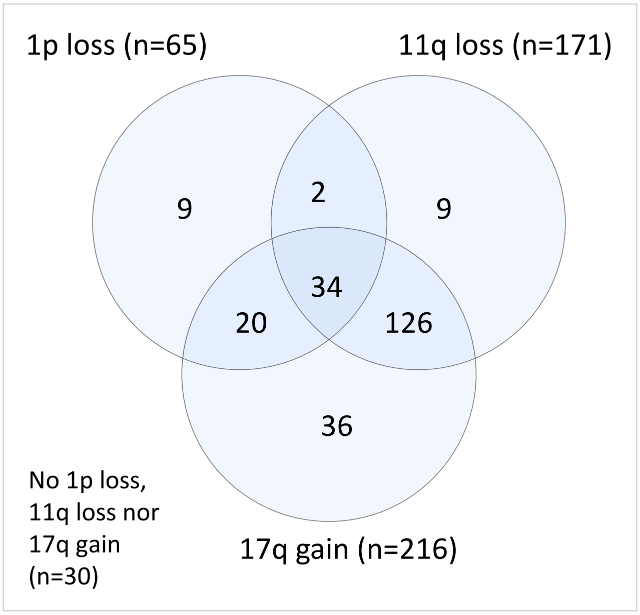

GG-P2 subtype (n=34) is accompanying 1pL,11qL, and 17qG.

Figure S3. Frequency of 1p loss, 11q loss, or 17q gain in 266 genetic "segmental" subtypes detected in *MYCN* non-amplified cases.
